# Potential and feasibility of preoptimisation in ventral hernia repair- a focus group study

**DOI:** 10.1007/s10029-025-03559-6

**Published:** 2026-01-16

**Authors:** Gunnar Nordqvist, Karin Strigård, Jeaneth Johansson, Catharina Gustavsson, Viktor Holmdahl

**Affiliations:** 1 Department of Diagnostics and Intervention, Umeå University, Umeå, 90185 Sweden; 2 Department of Social Sciences, Technology and Arts, Luleå University of Technology, Luleå, Sweden; 3 School of Business, Innovation and Sustainability, Halmstad University, Halmstad, Sweden; 4 Center for Clinical Research Dalarna, Uppsala University, Falun, Sweden; 5 Department of Public Health and Caring Sciences, Uppsala University, Uppsala, Sweden; 6 School of Health and welfare, Dalarna University, Falun, Sweden

**Keywords:** Hernia, Ventral Hernia, Incisional Hernia, Preoptimisation, Prehabilitation

## Abstract

**Aim:**

The aim of this study was to explore patients’ experiences of participation in a preoptimisation programme prior to ventral hernia repair, focusing on the programme’s feasibility and patient-perceived potential.

**Introduction:**

Preoptimisation of modifiable risk factors has received growing attention, but little is known about ventral hernia patients’ perspectives on such programmes. Understanding patient motivation and perceived barriers is critical for designing effective interventions.

**Method:**

Eight ventral hernia patients participated in a home-based preoptimisation programme lasting at least three weeks. Focus group interviews were conducted and analysed using reflexive thematic analysis.

**Results:**

Participants generally perceived the preoptimisation programme positively. Clear and structured advice was valued, with participants appreciating straightforward instructions on physical activity and lifestyle change. Reported barriers included everyday life circumstances, orthopaedic comorbidities, and in some cases the hernia itself. Patients described perceived positive health effects, including improved fitness, weight loss, and better diabetes control. No adverse effects were raised during the interviews.

**Conclusion:**

This is, to our knowledge, the first qualitative study of pre-optimisation programmes in ventral hernia surgery. As an exploratory, hypothesis-generating study, its findings are shaped by sample size and setting but provide novel insights that complement quantitative research by highlighting patient perspectives. Pre-optimisation programmes appear feasible and meaningful to ventral hernia patients, particularly when supported by clear guidance, structured follow-up, and surgeon involvement. The findings are discussed in the light of Self-Determination Theory, which emphasises internalisation of motivation through autonomy, competence, and relatedness.

## Introduction

Based on previous studies, an estimated 10–25% of patients develop an incisional hernia following laparotomy, of which approximately one-third require surgical repair. Complex ventral hernia surgery is associated with high risk for complications and recurrence [1]. Several factors that increase the risk for complications and recurrence have been identified [2, 3]. These include obesity, low level of physical activity, suboptimal treatment of underlying comorbidities, smoking, and alcohol consumption. These may all be treated or modified preoperatively: often referred to as preoptimisation or prehabilitation [4].

There has been growing interest in multimodal preoptimisation programmes targeting modifiable risk factors. However, while preoptimisation has been shown to decrease the number of severe complications in colorectal surgery, the positive effects of multimodal preoptimisation programmes in ventral hernia surgery remains uncertain. Furthermore, there are few studies on how preoptimisation programmes in ventral hernia surgery should be designed [4–7].While qualitative research in oncological surgery has shown that patients generally have a positive attitude towards preoptimisation [8–10], little is known of the attitudes of ventral hernia patients towards preoptimisation or how they experience participation in preoptimisation programmes. Patient attitudes include perception, feelings, and predisposition towards preoperative programmes, all of which can significantly influence their willingness to engage with and adhere to programme interventions. As motivation is a key factor in lifestyle changes, a deeper understanding of patient attitudes towards preoptimisation is vital when developing effective patient-centred preoptimisation programmes.

Self-Determination Theory, developed by Deci and Ryan (1980. 1985) [11], is frequently applied to understand and explain the patient’s motivation to make lifestyle changes. According to self-determination theory, motivation can be either intrinsic, where behaviour is inherently rewarding, or extrinsic, driven by external rewards. Extrinsic motivation exists on a continuum ranging from low to high degree of internalisation. Intrinsic motivation or a high degree of internalisation is associated with a greater chance of succeeding in and maintaining changes in lifestyle [12–15].

The aim of qualitative research is to collect and analyse non-numerical data to gain insight into the individual’s experiences, beliefs, attitudes, and motivation [16]. Data are typically gathered through interviews. In focus group studies, a small group of participants are interviewed together on a specific topic. In this way the research team can benefit from interactions between the interviewer and the participants as well as in-group interactions between participants [17]. Reflexive thematic analysis is a method used to find themes and patterns of meaning in qualitative data. Six steps to performing thematic analysis have been described by Braun and Clarke [18]. In reflexive thematic analysis the researcher’s role in analysing and interpreting the results is recognised and reflexivity rather than objectivity is emphasised [19].

The aim of this study was to explore patients’ experiences of participation in a preoptimisation programme prior to ventral hernia repair, focusing on the programme’s feasibility and patient-perceived potential.

## Method

### Design

An explorative qualitative design with data collection through focus group interviews (FGI) was chosen to study patient experience of participating in a home-based preoptimisation programme prior to ventral hernia surgery. The study was approved by the Swedish Ethics Review Authority (dnr: 2023-00714-01). After verbal and printed information, participants gave written consent prior to participating in the study. As this is a qualitative study focused on patient experience and feasibility no clinical outcomes were quantified. The Consolidated criteria on reporting qualitative research (COREQ) were followed [20].

### Setting

This study took place in the Norrbotten region of Sweden. This vast area lies in the far north and has a population density of only 2,6 inhabitants per square km [21]. Almost half of the population live in urban cities on the coast while the other half live in rural towns or small villages.

### Participants and authors

Participants were recruited at a preoperative appointment for their ventral hernia at the Gällivare and Sunderby hospitals in Norrbotten. Inclusion criteria were: (a) patient accepted for ventral hernia surgery; (b) hernia aperture width 4–8 cm; and (c) age > 18 years. Exclusion criteria were: a), inability to understand the Swedish language; b) very limited physical ability; and c) need for acute or subacute hernia repair.

Given the pilot, feasibility-focused nature of the study we aimed for 6–12 participants to form 1–2 FGIs. As a qualitative study, our focus was on exploring acceptability and practical implementation, not on quantifying outcomes. Ten consecutive eligible patients within the recruitment window were invited to participate of which eight accepted. Of the eight patients included, six were female. Age ranged between 36 and 77 years. All participants resided in the Norrbotten region, some in an urban environment and others in rural areas. All participants had a BMI ≥ 30 kg/m^2^ ranging between 30 and 48, on average they had two comorbidities with hypertension and diabetes mellitus being the most prevalent.

The first author was a 36-year-old male PhD student who was also a surgeon experienced in ventral hernia surgery at Gällivare hospital. He had prior experience as a researcher in the surgical field but no prior experience in qualitative methodology. A team of experienced researchers supervised his research: a 34-year-old male PhD in the field of hernia surgery; a 65-year-old female professor of surgery at Umeå University experienced in qualitative methodology and with a vast experience of ventral hernia surgery; a 55-year-old female professor of economy at the Luleå University of Technology, LTU, with a vast experience of qualitative research, including focus group studies; and finally a 62-year-old associate professor of physiotherapy with vast experience in abdominal wall rehabilitation and qualitative research methods.

### Preoptimisation programme

Through a study of the literature, three target areas for preoptimisation prior to ventral hernia surgery were identified and applied in this study: (a) weight loss, (b) increased physical activity; and (c) optimisation of comorbidity medical treatment and substance use e.g., nicotine and alcohol [4, 7].

A home-based approach was chosen to suit the populations of both rural and urban areas in northern Sweden.

To achieve weight loss, participants with a BMI ≥ 30 kg/m^2^ were instructed to use the smartphone app Lifesum^®^, designed to help the user keep track of consumed calories and aid weight loss through calorie restriction.

Physical activity interventions were designed to meet the World Health Organisation (WHO) recommendations for weekly physical activity i.e., 150–300 min per week of moderate intensity aerobic physical activity together with muscle strengthening activities at least two days a week [22]. Several suggestions for activities that increase the pulse were included. Participants were also given three suggestions for strength training: (a) a core strength programme initially developed for rehabilitation after diastasis recti surgery; (b) a video of bodyweight exercises published by the Swedish digital health service (www.1177.se) [23]; and (c) a gym-based resistance training programme for beginners published by the Swedish gym company Friskis&Svettis™ [24]. All participants were told to aim at 30 min of pulse-raising activities a day and one session of any of the suggested resistance training activities a day.

Optimisation of comorbidity medical treatment and nicotine and alcohol consumption were addressed at a digital appointment with a general practitioner linked to the study.

The participants were asked to follow the suggested preoptimisation programme for at least three weeks during the summer of 2023. Throughout the process, the first author followed the participants’ progress from inclusion to the end of the study through weekly phone calls aimed to identify problems and to motivate the patients. Short notes were made from these calls, tracking the progress of the participants as well as the researcher´s experience. The notes were used by the first researcher to help design the interview guide.

### Data collection

In August 2023, FGIs were held. The FGIs were moderated by the first author. This deliberate choice is congruent with reflexive thematic analysis [18, 19], which recognises that meanings are produced in researcher-participant interaction and that researcher position shapes, and can enrich, knowledge production [19]. The first author’s dual role as both treating surgeon and interviewer represented an “insider researcher” position [25]. While this facilitated trust, openness, and shared understanding with participants, it also introduced a potential risk of social desirability bias [26, 27]. To mitigate this, participants were informed that study participation would not affect their medical treatment, a non-clinical observer was present during interviews, and the interview guide was developed and revised in collaboration with experienced qualitative researchers. Regular preparatory and debriefing meetings with supervisors further supported reflexivity and minimised bias.

To make it possible for as many participants as possible to attend, two FGI occasions were offered. One participant, a 49-year-old female, failed to take part in any of the interviews. At the start of the interviews the first author held a short presentation of the subject and formal explanation of the interview process. The interviews followed a semi-structured outline of open-ended questions (Table 1). FGIs were held digitally in the form of video conferences using Zoom™. An administrator connected to the research group observed the interviews to further increase trustworthiness and to provide technical support if needed. FGIs were audio- and videorecorded and later transcribed by a medical secretary. The transcripts were 30 and 23 pages respectively.

**Table 1:** Interview guide

Overall Experience:
• What are your general thoughts about preoptimisation before hernia surgery? • Which part of the program did you find easiest? Which part did you find the hardest?
**Physical Activity**:
• How did you experience the parts of the program that focused on physical activity? • What kind of support would have helped you reach the physical activity goals? • What were your thoughts on the strength training program? • Did you try machine-based training at the gym? o What was that experience like? o Did the machines allow you to work your muscles more than the regular training program? • Did you try the exercise program from 1177.se? o What was that experience like? • What was your experience with the pulse-raising (cardio) activities? o Which activities worked well for you? Which ones didn’t? o What made it difficult to reach the activity goals? • How did you feel when the surgeon brought up physical activity before the operation?
**Weight Loss**:
• What thoughts or feelings came up when overweight and weight loss are discussed during a preoperative consultation? • What was your experience using the Lifesum^®^ app? o Was it difficult to get started? How could we have supported you better in getting started? o Do you feel that the app is a good tool for reducing calorie intake? • What kind of support would you have wanted to help lose 5–10 kg before surgery? • If there was a medication that could help make weight loss easier before surgery, would you consider using it?
**General Practitioner Visit**:
• How did you experience the digital doctor’s visit? • Do you feel that this type of consultation works well in a digital format? • Was there anything during the consultation that felt uncomfortable or difficult to talk about? • Do you think this is a good way to check for any untreated risk factors before surgery?
**Closing Questions About the Preoptimisation Experience**:
• What are your overall thoughts on preoptimisation before surgery? • Do you think it’s helpful when the surgeon brings it up? • Would you have preferred that modifiable risk factors were not considered before surgery? • Do you believe that preoptimization can affect the outcome after surgery?

### Data analyses

Reflexive thematic analyses following the principles described by Braun and Clarke were used [18, 19]. Data familiarisation began at the FGIs. Data were then transcribed verbatim and read through by all authors. The first author performed several rounds of inductive coding. Between rounds of coding, data were discussed with supervisors. Themes were developed in the later stages of coding and continuously revised. Naming of the themes was made with input from CLISTER (www.clister.se), a larger research group with abdominal wall surgery being one of its specialities. Data analyses were performed in Swedish, published quotes have been translated into English.

We applied reflexive thematic analysis [18], acknowledging that researcher subjectivity and positionality shape the analytic process [19]. Reflexivity was emphasised through ongoing dialogue between the insider (first author) and supervisors with outsider perspectives, supporting balanced interpretation and enhancing the credibility of findings.

## Results

The participants were generally very positive towards the concept of preoptimisation and the specific intervention programme they participated in. Patients were willing to have their doctor raise the question of preoptimisation at the preoperative visit and felt comfortable when talking about subjects such as obesity and low levels of physical activity in that setting. All participants were motivated to take part in the activities suggested. Barriers to succeeding with the programme included everyday life circumstances, major life events, and comorbidities. Two patients mentioned the hernia itself as being a hinder to successful preoptimisation. We generated eight themes from the analyses: positive attitude towards preoptimisation; contrasting feelings regarding own performance and ability; surgeon support as an important motivator; everyday life and comorbidities as barriers to success; individualisation facilitates the programme; positive effects of participation on health; easy-to-follow instructions on accessible interventions; and possible improvements to the programme.

- *Yes*,* I thought it was good. Of course*,* you should be informed that weight loss is beneficial since it reduces the risk for side-effects*,* or whatever you want to call it.*

Positive attitudes towards the preoptimisation.

All participants had a positive attitude towards the concept of preoptimisation. They all appreciated that questions on physical activity and weight loss were raised at the preoperative appointment. They expressed a feeling of empowerment in being able to take responsibility for their own treatment, possibly lowering the risk for complications.

- *I just felt it was very positive*,* like someone wanting to take care of me*,* setting certain expectations about what I could become good at myself*,* so for me it was only positive.*

Contrasting feelings towards own performance and ability.

The participants had mixed feelings regarding their own performances. However, everyone felt that they have managed to increase their level of physical activity.

- *Yes*,* it has gone well. I felt satisfied with myself at least.*
  *It has actually been a bit difficult to get started*,* I must say.*

Surgeon support as an important motivator.

All participants were highly motived to take part in the preoptimisation programme. They felt motivated to succeed with lifestyle changes to minimise the risk for surgical complications. They expressed the importance of receiving advice and help in achieving lifestyle changes through personal consultation rather than just being told they had to lose weight without getting any instructions. Close and recurrent follow-up, at least in the beginning of a preoptimisation programme, was perceived as important in increasing and maintaining motivation. Meeting others in the same situation was also mentioned as a motivator.

- *I was thinking about the other doctor I once spoke to*,* who just looked me up and down and said*,* ‘lose weight and come back later*,*’ without examining me or anything. I thought you (first author) were great*,* and that really motivated me. I also think about your follow-up phone calls (first author) during the first few weeks. Then it felt like - yes*,* this is important and that someone cares*,* and that contributed to me putting in a bit more effort.*

Everyday life and comorbidities as barriers to success.

Everyday life and comorbidities, especially orthopaedic comorbidities, were commonly mentioned as barriers to succeeding with preoptimisation. Two participants mentioned their hernia as being a barrier to keeping to the programme. Some of the participants thought it was harder to keep to the programme in the summer than it would have been the rest of the year.

- *Yes*,* I also have arthritis in a knee*,* and I have to say I haven’t been on all fours on the floor.*
  *Yes*,* I’ve been really bad at taking the time to do it since I’ve been very busy this summer*.
  *Yes*,* I’ve had so much pain in this upper abdominal hernia.*

Individualisation facilitates the programme.

Participants mentioned some factors that helped them overcome barriers and enable them to participate in the programme. Some made minor changes in the physical exercise regime to compensate for comorbidity. An elastic abdominal girdle seemed to help with hernia-related issues. One patient expressed that previous experience with physical activity helped.

- *F*,* 65: I have this in my knees*,* arthritis*,* that made me do everything … on the floor instead.*

Perceived positive effects of participation on health.

Participants experienced positive effects of participating in the programme. They mention improved fitness, improved strength, better glucose control, and better general health. No negative effects were mentioned.

- *Yes*,* what I have stuck to is walking*,* and I have taken different routes since I live in an urban environment. I have timed the distance I walk*,* sometimes one lap and sometimes two laps. At first*,* it took 30 min for one lap*,* and now I’m down to 25 min.*
  *Is there anyone else besides me who has sugar? First author: Have you noticed anything about your diabetes? Yes*,* I have because I had already shaped up before this programme*,* but it has gone down even more.*
  *I think I have started to feel much better*,* and the weight has gone down*,* so I think it’s only going in the right direction.*

Easy-to-follow instructions on accessible interventions.

Overall, the patients were happy with the programme they received. They wanted easy-to-follow instructions on accessible interventions. They were happy with the core-strength programme developed for rehabilitation after diastasis recti surgery. Poor access was a factor regarding weightlifting as well as difficulty in downloading the calorie restriction app, creating their profile, and obtaining premium access.

Opinions were mixed regarding the calorie restriction app. The app was considered too complicated by some, and they struggled to get started and to keep up with calorie registration. Others found the app to be a valuable tool to lower their daily calorie intake. Even those satisfied with the app would have preferred fewer functions. They were in general happy with the online general practitioner meetings. Some had difficulties with their online connection and were confused by the invitation. When it worked, the participants appreciated the digital form.

- *And this thing with Lifesum*,* keeping track of what I eat*,* I think has been great for me because I really have a good overview of the situation and have started to lose weight.*
  *Well*,* what I hear from the others is that they say it’s complicated*,* and there are many more features you have to keep track of that I find unnecessary.*
  *Yes*,* for my part*,* I experienced it like this when I was doing the exercises. They are easy to understand and not at all complicated to perform*,* so I thought*,* well*,* I’ll do this properly. And then that 1177*,* I haven’t even looked at it.*

Possible improvements to the programme.

The participants were generally very engaged in their programme activities. During the FGI, a will to take part in development of the preoptimisation programme was noted. The programme they followed was home-based, but during the FGI several participants appreciated the support of being part of a group. It was suggested that group activities with other patients in similar situation should be added to the programme in order to increase motivation. Some felt the need for better instructions, especially concerning how to use the app for weight loss but also concerning physical activity.

- *Yes*,* I would have wanted to have a group for people who are overweight. We could have exercised together.*
  *And also to be able to talk to*,* well*,* people who have the same problems as yourself*,* like hernias*,* for example… That would have been great. Like how we’re sitting and talking now*,* I think that’s fantastic.*

Participants were generally positive to the possibility of adding pharmacologically assisted weight loss to the programme. Most participants preferred the idea of orally administered glucagon-like peptide-1 receptor agonists (GLP-1 RAs) rather than subcutaneous injections. All participants were negative or hesitant towards bariatric surgery as part of preoptimisation.

- *Then you read about new pills that are aimed at diabetics*,* which can be used for weight loss and such*,* and that might be something*.

## Discussion

To our knowledge, this is the first qualitative study on preoptimisation in ventral hernia surgery. As a novel contribution, it provides unique knowledge about the attitudes of ventral hernia patients, how motivational factors interact to shape engagement as well as feasibility and potential of preoptimisation from a patient perspective. Designed as an exploratory and hypothesis-generating study, its conclusions should be further examined through both further qualitative and quantitative research. However, as the only study of its kind to date, the results should be considered in the design of patient-centred preoptimisation programmes.

This is a pilot study aiming to understand whether and how a multimodal, home-based preoptimisation programme can be introduced in routine ventral hernia care. We did not assess quantifiable clinical endpoints; reports of improved fitness, weight loss, or glycaemic control are self-reported and should be interpreted as perceived benefits that inform potential, not effectiveness. In this study the participants followed the program for at least three weeks which was considered sufficient to assess feasibility. Some participants followed the program for more than six weeks at the point of the FGIs. In preoptimisation prior to colon cancer surgery prehabilitation periods of 3–5 weeks have been tried with mixed results [28–31]. Given the benign nature of ventral hernias longer preoptimisation periods could be feasible if proven effective in quantitative studies.

Similar to what has been shown in oncological surgical settings [9], we found that patients demonstrate a generally positive and proactive attitude, with a strong willingness to take responsibility for improving their health outcomes, viewing preoptimisation as both relevant and beneficial. Applying Self-Determination Theory, motivation towards preoptimisation was mainly externally initiated(e.g., through surgeon advice and structured support), while, participants reflected a high degree of internalisation, which is associated with sustainable lifestyle change [32, 33].

Our findings underscore the critical need for active engagement and support from healthcare providers, particularly surgeons, to sustain patient motivation and ensure programme adherence. Regular follow-up, personalised guidance, and structured advice from the surgeon were perceived as highly motivating, as they not only provide patients with practical tools to succeed but also reinforced the perceived importance of the programme, thereby facilitating internalisation of motivation [14]. In contrast, generic advice, such as being told simply to “lose weight,” without guidance or follow-up, was perceived as discouraging and less effective. This may leave patients with a sense of incapability, which according to self-determination theory hinders internalisation of motivation and reduces programme effectiveness [14].

The participants in our study took part in a home-based preoptimisation programme. It was suggested that group training sessions and peer interactions could be a motivator. In fact, some participants expressed that the focus group interview itself provided motivation to continue with the changes in lifestyle they had achieved. This aligns with previous qualitative studies on preoptimisation and rehabilitation in surgical settings [8, 34], as well as in studies on medical conditions such as diabetes [35]. Again, in self-determination theory, being able to relate with others is said to facilitate internalisation of external motivation [14]. Future preoptimisation programmes could explore ways to integrate peer support, even in home-based designs.

Adherence to the programme was hindered not only by everyday life circumstances such as daily responsibilities and significant life events but also health related barriers. Orthopaedic comorbidity such as arthritis limited participation in physical activity while some participants found the hernia itself directly restrictive. This created the paradox that the condition targeted by preoptimisation could itself hinder engagement for the target group of patients with abdominal wall limitations. These findings highlight the need for condition-adapted exercise strategies, supportive devices, and individual tailoring. All participants, including those experiencing hernia-related limitations, reported that they were able to engage in the majority of the programme. Limitations in physical activity are a commonly reported concern among ventral hernia patients and often cited as a reason for seeking surgical treatment. Against this background, our findings are encouraging, as they suggest that some level of physical exercise is often feasible despite these challenges. The muscle-strengthening programme applied in this study was originally developed by an experienced physiotherapist for rehabilitation after rectus diastasis surgery and is specifically designed to be adapted to a limited abdominal wall. We suggest that core exercises adapted for patients with limited abdominal wall function, focusing on individual ability, are applicable to ventral hernia patients and may represent a suitable approach to abdominal strengthening in preoptimisation programmes. Future research is needed to learn more about hernias as a barrier to physical exercise, adaptions in physical activity to meet the needs of hernia patients and if supportive devices such as abdominal binders is helpful in enabling physical exercise.

Patients participating in preoptimisation programmes mainly described perceived health benefits, including, weight loss, increased physical strength, and significant lifestyle changes, even when adherence to the programme is only partial. Negative consequences were not raised in the interviews, though this does not exclude the possibility of unexpressed or unrecognised challenges. Note that any quantification of health benefits, clinical outcome and adverse effects is outside of the scope of this qualitative study and only participants’ experiences are reported. Future studies should therefore investigate both benefits and potential burdens of preoptimisation. The negative effects of preoptimisation also require quantitative investigation, since one previous study showed a tendency towards higher risk for emergency surgery in patients undergoing preoptimisation [5].

Participants were generally reluctant to consider bariatric surgery as part of preoptimisation, which may challenge staged approaches sometimes recommended for severely obese hernia patients with a complex ventral hernia [36]. Clinicians should be aware that some patients in this study expressed skepticism towards bariatric surgery as part of preoptimisation. Such attitudes could potentially influence motivation to engage with other components of the programme. On the other hand, pharmacological assistance such as GLP-1 RAs were viewed positively by participants. In a recent study, the use of GLP-1 RAs in combination with dietary advice was shown to accelerate weight loss when compared with preoperative dietary advice only in ventral hernia patients [37]. This finding suggest that the broader use of GLP-1 RAs could possibly enhance preoptimisation effects and that ventral hernia patients could be motivated towards such treatment. These insights indicate that incorporating GLP-1 RAs into preoptimisation programmes could represent a promising way to strengthen patient engagement and outcomes. However, further research is needed to evaluate the feasibility, long-term safety, and cost-effectiveness of integrating pharmacological interventions alongside lifestyle-focused preoptimisation.

Participants expressed a clear preference for a straightforward and manageable preoptimisation programme. Complex interventions or technical tools were less appealing and harder to deal with. Digital tools such as Lifesum™, and prerecorded resistance training videos proved challenging for many patients. Similar results have been shown in preoptimisation in thoracic surgery and telerehabilitation in stroke patients [38, 39]. We recommend that preoptimisation programmes should prioritise accessible, stepwise, and patient-friendly approaches, supplemented by support and tuorials where digital tools are used. Future research should explore how digital interventions can be simplified, personalised, and integrated with professional support to improve usability and adherence in ventral hernia patients.

Limitations and suggestions for future research.

This study has several limitations. Small sample size and female predominance may limit generalisability. Furthermore, the geographical context should be considered when interpreting findings. The study may be affected by selection bias, as all participants volunteered to participate in preoptimisation. However, only two patients declined participation, both citing that they already considered themselves fit and healthy. Future interview studies with ventral hernia patients who are unwilling or feel unable to participate would be of great interest to further explore motivational factors and barriers in this group. A deliberate methodological choice was made to let the surgeon who followed the participants through the preoptimisation period act as interviewer and main developer of themes, in line with reflexive thematic analysis [19]. This insider role provided shared experiences, pre-existing trust and shared language between clinician and patients that facilitated a richer and more nuanced discussion, especially of personal and sensitive matters. In this way, insider researchers can gain access to knowledge that improves data quality as well as contextual interpretation that might be missed by outsider researchers. However, this methodology comes with a risk of bias. We acknowledge the risk that our results to some extend might be influenced by power dynamics between the first author and the participants and by social desirability providing responses they believed would please the interviewer. To mitigate this risk, participants were informed at the start of focus group interviews that results from the study did not affect their medical treatment, a non-clinical observer was present during focus groups, a semi-structured interview guide revised by experienced supervisors was used and the first author and a non-clinical supervisor with vast experience of qualitative research conducted preparative meetings before focus groups and debriefings afterwards.

We emphasise the need for further research, both studies that quantify the benefits and validate outcomes reported in this qualitative study. Additional qualitative studies are also warranted to confirm or challenge our results and to refine programme components, including exploring interventions that may enhance effectiveness. We suggest future research to evaluate the short- and long-term effects of structured preoptimisation programmes on modifiable risk factors, surgical complications, and overall outcomes. Randomised studies incorporating patient feedback and simplified interventions will be essential to ensure accessibility and effectiveness.

Findings from this qualitative study provided the foundation for the development of a structured, patient-centred preoptimisation programme. This programme is currently being evaluated in a randomised controlled trial comparing it with standard clinical practice to establish its impact on patient outcomes.

## Conclusion

This study suggests that preoptimisation in ventral hernia care is both feasible and meaningful to patients, particularly when supported by structured advice, surgeon follow-up, and opportunities for tailoring to individual limitations. While the study is limited in scope, it highlights important directions for future research, including evaluating programme effects in larger and more diverse populations, and assessing long-term outcomes of preoptimisation. As some patients acknowledged the hernia itself as a barrier towards physical preoptimisation further research on condition specific adaptations are also warranted. The findings are discussed in the light of Self-Determination Theory, with autonomy, competence, and relatedness emerging as central psychological needs that can guide the design of effective, motivating preoptimisation programmes.
